# A novel expert system for objective masticatory efficiency assessment

**DOI:** 10.1371/journal.pone.0190386

**Published:** 2018-01-31

**Authors:** Gustavo Vaccaro, José Ignacio Peláez, José Antonio Gil-Montoya

**Affiliations:** 1 International Postgraduate School, School of Dentistry, Granada University, Granada, Spain; 2 Department of Languages and Computer Sciences, University of Malaga, Malaga, Spain; 3 Prometeo Project, National Secretary of Higher Education, Science, Technology and Innovation (SENESCYT), University of Guayaquil, Guayaquil, Ecuador; 4 Gerodontology Department, School of Dentistry, Granada University, Granada, Spain; University of Toronto, CANADA

## Abstract

Most of the tools and diagnosis models of Masticatory Efficiency (ME) are not well documented or severely limited to simple image processing approaches. This study presents a novel expert system for ME assessment based on automatic recognition of mixture patterns of masticated two-coloured chewing gums using a combination of computational intelligence and image processing techniques. The hypotheses tested were that the proposed system could accurately relate specimens to the number of chewing cycles, and that it could identify differences between the mixture patterns of edentulous individuals prior and after complete denture treatment. This study enrolled 80 fully-dentate adults (41 females and 39 males, 25 ± 5 years of age) as the reference population; and 40 edentulous adults (21 females and 19 males, 72 ± 8.9 years of age) for the testing group. The system was calibrated using the features extracted from 400 samples covering 0, 10, 15, and 20 chewing cycles. The calibrated system was used to automatically analyse and classify a set of 160 specimens retrieved from individuals in the testing group in two appointments. The ME was then computed as the predicted number of chewing strokes that a healthy reference individual would need to achieve a similar degree of mixture measured against the real number of cycles applied to the specimen. The trained classifier obtained a Mathews Correlation Coefficient score of 0.97. ME measurements showed almost perfect agreement considering pre- and post-treatment appointments separately (κ ≥ 0.95). Wilcoxon signed-rank test showed that a complete denture treatment for edentulous patients elicited a statistically significant increase in the ME measurements (*Z* = -2.31, p < 0.01). We conclude that the proposed expert system proved able and reliable to accurately identify patterns in mixture and provided useful ME measurements.

## Introduction

### Objective evaluation of the masticatory function

Health care services for the elderly and physically disabled population are ever-increasing challenges where practitioners are required to evaluate the functional impairments of individuals in faster and more accurate ways while using less invasive methods. One approach to this matter is the objective evaluation of the human mastication, which is a complex biomechanical process that involves coordinated movements of the jaw, tongue, lips, and cheek; and one of the main functions of the stomatognathic system. [1].

Objective mastication assessment can be performed in two ways: firstly, by quantifying the changes that the food has suffered during mastication, i.e. the Masticatory Performance; and secondly, by calculating the number of chewing strokes that would be required to achieve a certain degree of food degradation, i.e. the Masticatory Efficiency [2]. The Masticatory Performance (MP) has been defined as: a measure of the comminution of food attainable under standardized testing conditions [3]; and is considered an objective indicator of oral functional capabilities, widely used to measure the impact of dental treatments, to assess levels of disability and orofacial functional impairments following stroke [4,5], and has also been associated with malnutrition risk [6]. On the other hand, the Masticatory Efficiency (ME) has been originally defined as the number of extra chewing strokes needed by the patient to achieve the same pulverization as the standard person [7]; however, the strict measurement of the ME is presently in disuse, mainly because patients with impaired mastication would need to masticate for very large periods of time. Furthermore, it is important to notice that several studies used the terms MP and ME interchangeably while referring exclusively to the MP.

Current MP assessment techniques are based on the objective quantification of the degradation of a test-food subjected to mastication. The degradation level is determined by measuring a property (colour, weight, median particle size, chemical concentration, etc.) of a piece of natural or artificial food (e.g. Optosil/Optocal^TM^, peanuts, ham, chewing gums, paraffin blocks, carrots, jelly gums, etc.), where the property is prone to changes related to the number of chewing strokes.

**Fig 1:**
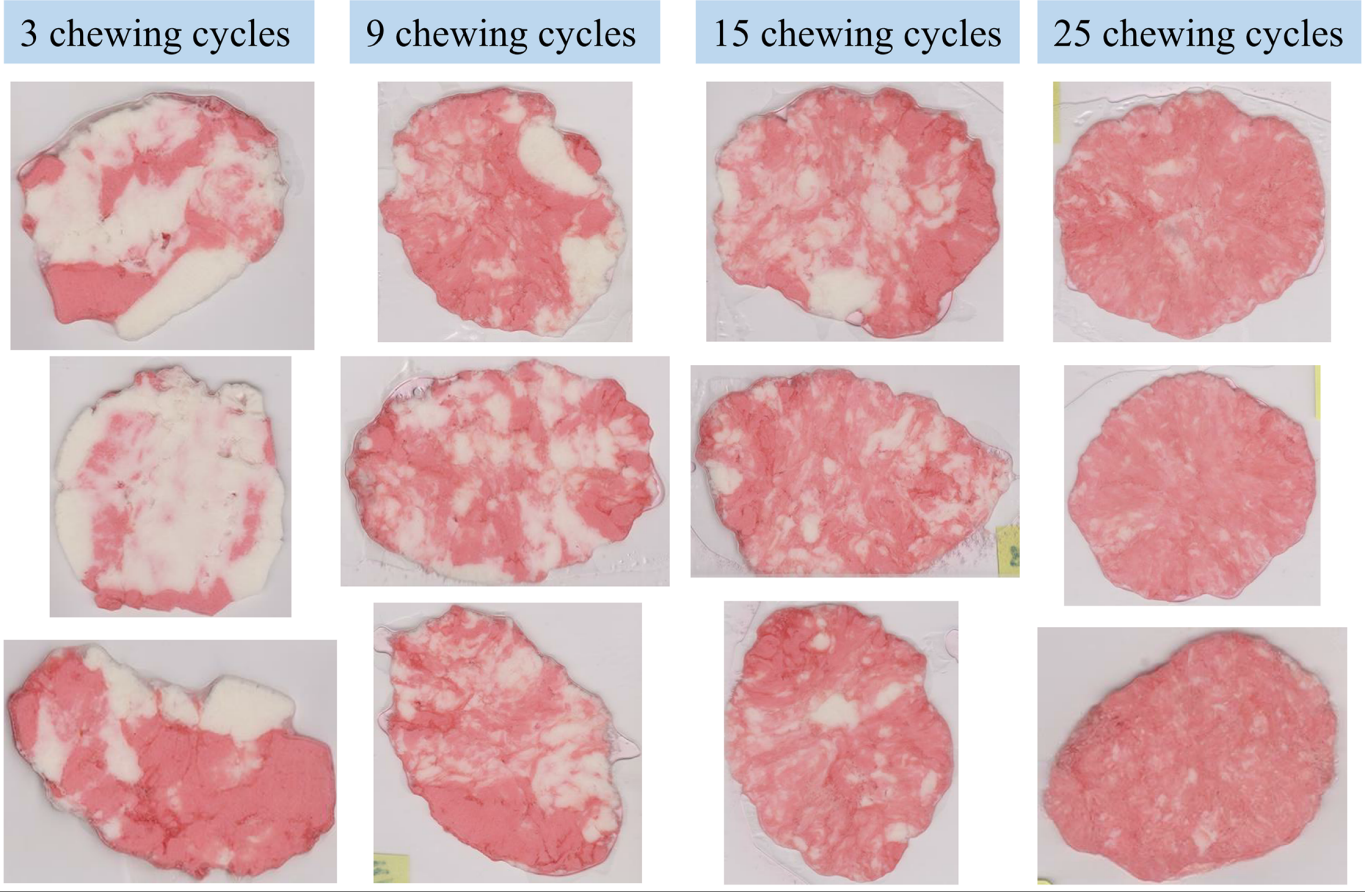
Example set of masticated chewing gums.

The fastest and easiest routine for objective MP assessment is the mixture quantification of a two-coloured cohesive specimen subjected to mastication [8–10]. In a mixing test a test-food specimen is formed by two differently-coloured layers of chewing gum or paraffin stacked together. Previous studies suggest that there are similarities among the visual characteristics of chewing gums masticated for the same number of chewing strokes when considering young and healthy human subjects [11]. These similarities have allowed experts to subjectively identify the mixture using comparison tables. An example set of masticated specimens for 3, 9, 15, and 25 chewing cycles is shown in Fig 1; where it is possible to notice that the red and white layers are mixed, to some extent, in a regular fashion. The amount of mixture reached with each chewing cycle would depend on the masticatory function of the individual and on the structural characteristics of the specimen such as the size, thickness, density, hardness, viscosity, and tinctures used for colouring.

Several studies have proposed simple digital image analysis approaches for mixture quantification that are more precise than visual inspection techniques, and modern mixing tests currently focus on these kinds of procedures [8]. The first attempt to measure the mixture of food using digital image analysis employed several custom-made algorithms, but these were not fully described, hence not possible to replicate [12]. Later on, the *magic wand* tool of the Adobe Photoshop Elements® software was used to select and count the pixels corresponding to the regions of the masticated bolus that were not mixed [8]. The “unmixed fraction” of the specimen was manually calculated using a Microsoft Excel® spreadsheet (Microsoft Corporation, One Microsoft Way, Redmond, WA, USA). The measurements provided by this procedure were highly affected by the user, meaning high variability of the results; also, it was time-consuming and difficult for clinical settings.

These difficulties promoted the creation of a specialized tool called ViewGum (dHAL Software. Kifissia, Greece, www.dhal.com), which has been receiving special attention from the dental and medical communities because of its ease of usage [9,13]. ViewGum uses the Bai and Sapiro segmentation algorithm to isolate the bolus from the background of the image, and measures the Circular Standard Deviation of the Hue channel (SDHue) in the HSI colour space; however, SDHue measurements may not to be suitable for white-coloured chewing gums [9,11], thus limiting the range of potential test-foods. In a different work, the Wolfram Mathematica® software (Wolfram Research, Champaign, IL, USA) was used to compute the custom visual feature “DiffPix” for mixing quantification [10]; nevertheless, neither the segmentation, nor filtering, nor the feature extraction procedure itself were clearly exposed, so the DiffPix computation approach is not available for reproduction.

### The problematics of masticatory performance assessment

The MP quantification techniques based on digital image processing retain various weaknesses. Firstly, most of the algorithms or tools employed for this task are not well-documented [8,9,11–13]. Also, there is neither consensus about which properties of the test-food should be monitored, nor scales for the mixture. The mixture can be quantified by numerous features of the resultant bolus [9,11,14]; however, existing MP measurement approaches rely on a single feature as the MP indicator, thus leading to high variability in the measurements and severely limiting the assessment methodology to a narrow set of test-foods [11]. Furthermore, MP assessment techniques require specialized training, equipment, and considerable amounts of time. For all these reasons, a comprehensive and integral reference framework for assessing the mixture in two-coloured chewing gums is needed.

### Proposed approach and purpose of this work

This study explores the possibility to accurately measure the level of mixture in two-coloured chewing gums subjected to mastication within a comprehensive reference scale (0 to 100%). Consequently, important questions arise: what aspect of the masticated bolus should be considered as the mixture indicator? And, from other point of view: how do specimens mixed by 0%, 25%, 50% (and so on) look like? These are not easy questions, as there are limitless ways to describe a digital image; and on the other hand, mastication is an erratic process, thus samples mixed under similar conditions would surely present noticeable differences.

To overcome these difficulties, this study redefines the MP and ME under the premise that it is possible to identify patterns in the visual characteristics of masticated two-coloured chewing gum specimens when considering a young and healthy reference population. Firstly, we have redefined the MP for mixing tests as “the set of measurements that characterize the state of a sample subjected to a given number of chewing strokes”, thus extending the original definition to include more than one feature. On the other hand, the MP of a given specimen would not suffice to achieve a diagnosis of the masticatory function of the patient, because a reference dataset is needed for comparison. Therefore, we have also redefined the ME for mixing tests as “the equivalent number of chewing strokes that an individual from a healthy reference population would need to achieve a similar degree of mixture under controlled experimental conditions measured against the known number of chewing strokes applied to the sample”. The key aspect of this new ME definition is the calculation of the number of chewing strokes that would produce a *similar* MP outcome for the reference population; therefore, the challenge of evaluating the masticatory function of an individual can be summarized as a classification problem where the MP (observed state) of a masticated specimen is classified into a category represented by the number of chewing strokes needed by the reference population. Mathematically, the ME can be represented as:

$$ME=\frac{P}{T}$$ (1)

where ME ≥ 0, *P* is the number of chewing strokes needed for an individual from a healthy reference population (*P* ∈ **N**,), and *T* is the known number of chewing strokes applied to the sample (*T* ∈ **N**, *T* > 0). Consequently, a specimen with a ME of 0 implies a total absence of mixture, while a ME of 1 implies a normal level of mixture, i.e. comparable to what a reference healthy person would achieve. Furthermore, an ME > 1 implies that the diagnosed individual chews better than the average healthy person. The ME can also be expressed as a percentage for easier interpretation and easily associated with linguistic tags as shown in Table 1.

**Table 1:** Example of linguistic tags associated to Masticatory Efficiency levels.

Linguistic tag	Masticatory Efficiency level
Totally impaired	0%
Impeded	25%
Limited	50%
Adequate	75%
Normal	100%
*Better than the norm*	Greater than 100%

The issue of evaluating multiple visual characteristics and performing an accurate classification of the sample can be efficiently solved by the application of computational intelligence techniques for pattern identification and automatic classification. Therefore, the aim of this paper is to present and validate a novel expert system for mixture patterns recognition in young and healthy individuals, and objective Masticatory Performance efficiency assessment in masticatory-compromised individuals. The hypotheses tested in this work are:

1. The proposed system can accurately classify masticated two-coloured chewing gum specimens into the corresponding group represented by the number of chewing cycles applied.
2. The proposed system is able to identify differences between the patterns of mixture of edentulous individuals prior and after treatment with complete removable dentures.

## Materials and methods

### Knowledgebase

**Fig 2:**
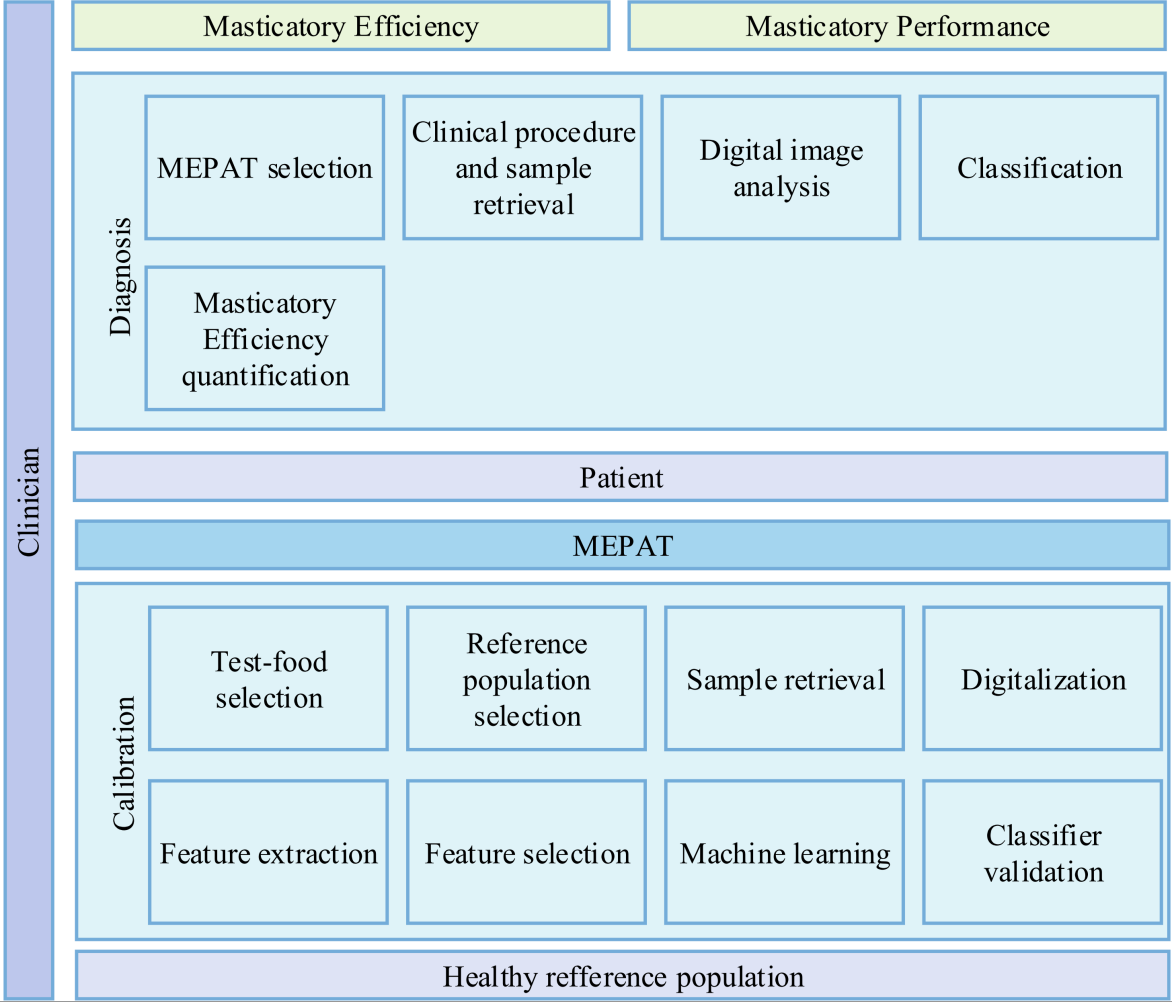
Proposed mastication assessment solution model.

The proposed expert system involves the identification of mixture patterns in two-coloured chewing gums; these patterns are the basis of a classification procedure to compute the *P* score (see Eq 1) of new specimens obtained from masticatory-compromised individuals. However, it is important to notice that the structural characteristics of a chewing gum brand are related to its visual characteristics; besides, a brand’s availability may not be the same worldwide. To overcome these problems the proposed system comprises two main components, as shown in Fig 2: a calibration stage oriented to identify patterns in the mixture of a reference population for a selected chewing gum brand; and a diagnosis stage oriented determine the ME of a patient using the same brand of chewing gums. These are related by an auxiliary component called Masticatory Efficiency and Performance Assessment Technique (MEPAT) which contains the information generated from the calibration in order to accurately perform masticatory assessment tests.

### Calibration

The calibration stage aims to identify patterns in the visual characteristics of masticated two-coloured chewing gums by analysing a broad distribution of reference samples. This process can be resource-intensive and time-consuming because it requires the participation of various reference individuals and the analysis of multiple samples per participant. In this work, the calibration stage was performed in the Faculty of Dentistry of the University of Guayaquil, Ecuador; and was orchestrated by four trained clinicians. The calibration process comprises the test-food selection, reference population selection, sample retrieval, digitization, segmentation, feature extraction, feature selection, machine learning, and classifier validation steps; which are described in the following sections.

#### Test-food selection

The test-food considered for this approach was a chewing gum wafer composed of two differently coloured layers. These colours were different enough to permit an adequate interpretation of the level of mixture with a mere visual inspection. Previous studies have proposed red-blue[8], green-blue [13], red-white [11], among other colour combinations. In this work, the selected test-food was composed of two flavours of Trident® chewing gums: watermelon (red dye) and spearmint (white dye); which are commercially available in Ecuador at the time of the experiment. The chewing gum strips came in the form of individually wrapped strips measuring 2.5 × 9 × 38 mm. Each specimen was formed by manually stacking the two pieces of chewing gum.

#### Reference population selection

A total of eighty volunteers (*N*_*1*_ = 80) were recruited for the reference group (G_1_): 41 females aged 25 ± 4.2 years; and 39 males aged 25 ± 5.8 years. Subjects were students from the Faculty of Dentistry of the University of Guayaquil, Ecuador. The inclusion criteria were being 18 to 35 years old, having at least 28 natural teeth, Angle Class I occlusion, and a DMFT score of 2 or less. Exclusion criteria were TMJ dysfunction symptoms, orofacial pain, bruxism, tooth wear, and the presence of fixed or removable orthodontic appliances. Written informed consent was obtained from all participants. Formal approval through the Ethical Committee for Human and Animal Experimentation of the University of Guayaquil was obtained for this experiment.

#### Sample retrieval

An operator instructed the subjects in G_1_ to masticate five specimens by 0, 5, 10, 15, 20 chewing cycles correspondently, and silently counted the number of cycles. Subjects rested for 30–60 seconds between mastication sessions to prevent fatigue. The subjects expelled the resultant boluses and the operator located each bolus between two sheets of transparent film intended for document lamination, and immediately flattened them to a 1mm thick wafer using a wheel-driven screw press. The flattening step is important because this assembly provides resistance to manipulation and is ideal digitization [9]. A total of 400 samples were collected this way.

Regarding the selection of the set of chewing cycles, previous studies indicate that this list must include 20 chewing strokes as the mean human mastication duration; and no more than 50 chewing strokes because of fatigue of the masticatory muscles [15]. On the other hand, 0 chewing strokes represents the “pre-mastication state” of the specimen, and it is obtained by retrieving the food specimen right after the subject introduces it into the mouth. This is important because saliva may play an important role during the image analysis process. Nevertheless, the task of selecting the adequate number of chewing cycles is currently a point of discussion among scholars.

#### Digitization

All specimens were individually scanned on both sides using a Canoscan Lide 220® flatbed scanner (300 dpi, standard calibration parameters for colour images). A flatbed scanner was chosen against digital photography because empirical experimentation showed that scanned images offered better image quality and even illumination. However, empirical tests during the calibration stage exhibited that digital photography under controlled conditions may also provide adequate images.

#### Segmentation

The resultant digital images were segmented to isolate the area of the bolus against the background. Specimens often had irregular shapes, vague boundaries, and very heterogeneous coloration; therefore, these conditions made precise automatic segmentation a nontrivial task. Active contour modelling have been used previously, but required additional effort for the clinicians [13]; so a more general approach was needed. Watershed algorithms can serve to transform the brightness levels of the image into a set of clusters or “*water pools*” by identifying relevant markers on the image and filling their surroundings [16]; in this regard, improvements of the Watershed algorithm has been proposed in the literature which are specially tailored for complex medical imaging [17]. On the other hand, Watershed algorithms can become highly complex for colour image segmentation and usually identify many clusters per image, hence they often require post-processing methods. Another approach for this matter involves iterative shrinkage methods for the selection of a specific region in the image, which has been successfully used for complex medical imaging segmentation such as magnetic resonance imaging [18]; however, these are better suited for sparse level images with diffuse contours and may require supervised intervention to indicate the desired search region.

**Fig 3:**
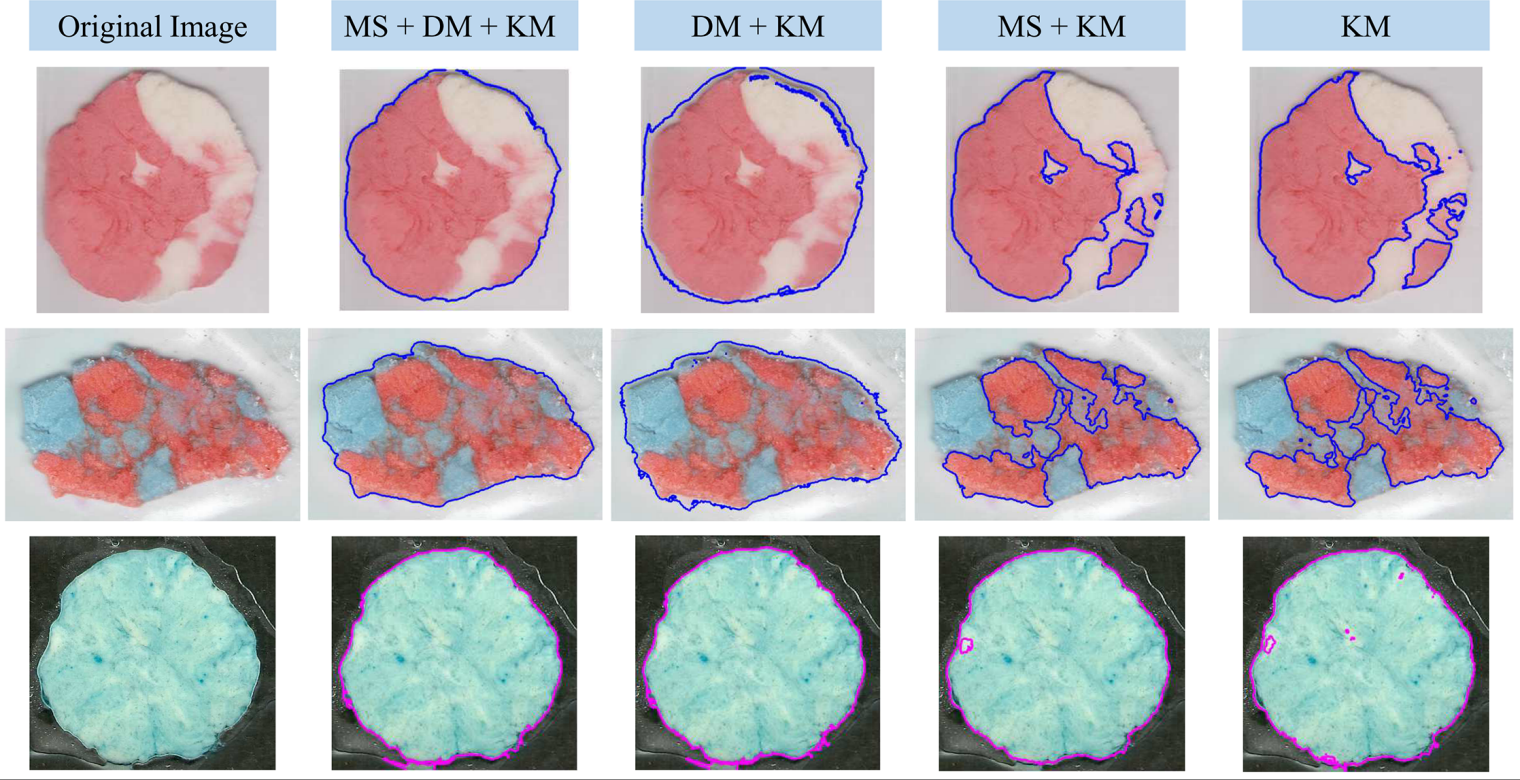
Comparison of different automatic segmentation methods applied over chewing-gums identification. The goal of the segmentation process was to discriminate the chewing gum bolus in the centre of the image against the background. Three segmentations methods were employed: Mean Shift (MS), Distance Map (DM) and K-Means (KM).

In this regard, the proposed approach implemented a fully-automated colour-based segmentation algorithm, constructed upon the combination of Mean Shift [19,20], distance map, and K-Means classification algorithms [21]. Further details about this segmentation approach are detailed in S1 Appendix. This custom-made segmentation algorithm was used to divide the image into two regions: the bolus located in the centre of the image, which is considered as the Region of Interest (ROI); and the Background, surrounding the bolus. An example of the application of the proposed region classification process considering Mean Shift (MS) + distance map (DM) + K-Means (KM) is shown in Fig 3, where it is possible to notice an improvement in the classification of regions, compared to the usage of KM, MS + KM, and DM + KM.

#### Feature extraction and MP calculation

The proposed expert system approach considers the whole observed state of a specimen, i.e. the MP of the sample. Mathematically, the MP can be described as:

$$MP=(f_{1},f_{2}\ldots ,f_{k})$$ (2)

where f_*i*_ represents the *i*^th^ observed feature, and *k* represents the total number of features. It is important to notice that if just one feature is considered then it may lack of sufficient information about the sample; consequently, the MP comprises multiple features at once. Additionally, these features can be obtained from different extraction methodologies that are already acknowledged by clinicians, e.g.: pixel counts, histogram analysis, etc. [10,13]. Under this premise, a custom set of feature extraction models was applied over the ROI of each pair of images corresponding to the same specimen. Feature extraction models included the mean and the absolute variance of the colour pixels; and the absolute variance, skewness, energy, entropy, and highest peaks of the colour histograms. These were computed for each component of the RGB, CIE-L*u*v* [22], HSI, and Normalized RGB colour spaces separately (see S2 Appendix). Additionally, the Circular Variance of the Hue channel of the HSI colour space was computed [9,13]. Consequently, the feature extraction stage considered a total of 121 features obtained from different image processing methodologies and colour space models (10 methods × 12 channels + *CVOH*). Heuristic extraction models such as Binary Particle Swarm Optimization or advanced texture characterization like Wavelet-based methods were not considered for this experiment because of their high computational times compared to simple pixel and histogram feature extraction models; nevertheless, further improvement of the proposed solution should make extensive use of these kind of advanced characterization approaches [23,24].

The selection of the colour spaces was based on empirical experience. In this case the original images are represented in the RGB colour space, which is used for fast representation of 256 shades of Red, Green, and Blue. The CIE-L*u*v* is an easily computable transformation of the CIE XYZ (Tristimulus) colour space [25], extensively used in graphical computing. The HSI (Hue, Saturation, and Intensity) colour space is a cylindrical-coordinate representation of the points in the RGB with applications in computer vision [24], and previous studies suggest it is useful for characterizing the mixture of chewing-gums [9]. Finally, the Normalized RGB colour space is obtained from the RGB by a normalization procedure [26]; the influx of the brightness is diminished by the normalization, so it is less susceptible to changes related to the light-source.

#### Feature selection

The large number of features increases the chances of accurately characterizing a sample; however, it hiders the effectiveness of pattern recognition processes that will be applied in further stages. It is possible to discard some of the features by computing a relevancy score (*q*) associated to each extracted feature, computed as:

$$q_{i}=\left(\frac{\left|\rho (F_{i})|+(\frac{\gamma (F_{i})}{\left(\begin{array}{c} n\\ 2 \end{array}\right)}\right)}{2}\right)$$ (3)

where F_*i*_ represents the set of measurements obtained from extraction of the *i*^th^ feature, *ρ*(F_*i*_) is the coefficient of correlation with the number of chewing cycles calculated with Spearman’s rho, *γ*(F_*i*_) is the amount of statistically different pairs of chewing strokes that the *i*^th^ feature can discriminate, and *n* is the amount of different numbers of chewing strokes (*n* = | ***C*** |). The value *γ*(F_*i*_) is calculated with ANOVA, considering the number of chewing strokes as the fixed factor, corrected with post hoc Bonferroni, and considering that:

$$0\leq \gamma (F_{i})\leq \left(\begin{array}{c} n\\ 2 \end{array}\right)$$ (4)

A feature can be discarded if the associated *q* score is lower than 0.5. The above-mentioned feature selection process may serve to reduce the size of the Artificial Neural Network model that will be used for pattern recognition in this experiment. Additional dimensionality reduction can be achieved by applying principal component analysis (PCA) to the set of non-discarded features by converting the set of possibly correlated features into a set linearly uncorrelated principal components [27]. Nonetheless, previous experimentation showed that dimensionality reduction using PCA may not necessarily improve pattern recognition performance of this solution. In this regard, PCA results for the non-discarded features are shown in the Results section for comparison purposes.

#### Machine learning

The information extracted from each specimen (S) during the calibration phase was summarized as a 2-touple consisting of the MP of the specimen and the number of chewing strokes (*t*), such that:

S=(MP,t) (5)

Then, the specimens were grouped as:

$$S_{i}=\{S|S=(MP,x)\wedge x=t_{i}\}$$ (6)

where **S**_*i*_ represents the set of MPs of all specimens that were masticated by *t*_*i*_ chewing strokes.

The proposed methodology used an artificial neural networks (ANN) algorithm for pattern identification [28–31]. ANNs are rough mathematical models of biological neurons where electrical signals are represented as numerical values; they mimic some features of biological brains, especially the ability to learn, i.e. to acquire the ability of processing information in certain patterns [32]. The chosen ANN architecture was the Multilayer Perceptron (MLP) [33]. The MLP maps a set of inputs to a set of desired outputs through multiple layers of nodes; where each node is an artificial neuron. The basic MLP structure consists of an Input layer, an Output layer, and any number of Hidden layers [24,34–36].

In our case, the MLP structure consisted of *k* nodes in the Input layer (where *k* is the number of features extracted from the specimens), 1 node in the Output layer (binary: true or false), and a variable number *h* of nodes in the Hidden Layer (*k*/3 ≤ *h* ≤ *k*). A set of MLPs was constructed for each **S**_*i*_ with the task of determining if the sample was masticated by *p*_*i*_ chewing strokes and considering different numbers of hidden neurons. The best number of neurons *h* in the Hidden layer was computed by sequentially increasing by 1 the value of *h* from *k*/3 to *k*, and executing 10 trainings per *h* value.

Inputs and outputs were obtained by breaking the information (i.e., the S) of each sample in two: the MP was considered the input, and the *p* as the output. The entire data set was randomly divided in three groups for each training execution: 40% for the Training Group (TG), 30% for the Validation Group (VG), and 30% for the Testing Group (SG). Each MLP was trained using the data in the TG, and using the VG as a reference to stop overfitting [37].

#### Classifier validation

The proposed system fed each trained network with the MP inputs from the SG group, and compared the predicted outputs (the *P* score) against the known number of chewing strokes. Then, the system computed the Matthews Correlation Coefficient (MCC) to assess the performance of a trained network for each execution [38]. The MCC is a measurement of the quality of binary classification that considers true and false positives and negatives, and can be regarded as a correlation coefficient between the observed and predicted classifications, where MCC = 1 represents a perfect prediction, MCC = 0 represents a random equivalent prediction, and MCC = -1 represents a complete disagreement between prediction and observations. The MCC was computed as follows:

$$MCC=\frac{TP\times TN-FP\times FN}{\sqrt{\left(TP+FP)\times (TP+FN)\times (TN+FP)\times (TN+FN\right)}}$$ (7)

where TP represents the number of true positives, TN represents the number of true negatives, FP represents the FP represents the number of False Positives, and FN represents the number of False Negatives. The system considered an MPL as suitable if its MCC was over 0.95, and choose the MLP with the highest MCC. The calibration stage would be considered unsuccessful if no suitable MLPs are found after iterating through all the possible *h* values. The system assembled the set of suitable MPLs for each *p*_*i*_ as a unique classifier in the form of a binary cascade: new samples were classified for **S**_*1*_, then for **S**_*2*_, and so on. The system discarded any sample that could not be classified into any **S**_*i*_ group.

### The MEPAT

The information obtained from the calibration stage includes details about the Test-Food, the features that best characterize it, relevant data about the experimental procedure, and the trained classifier. To perform diagnostic analyses over new specimens a clinician operator must follow the same sample retrieval procedure, utilize the same feature extraction methods, and feed the trained classifier with information about a new sample formatted in the right way. This paper proposes a new standardized representation of the information, procedures, and tools required to perform Masticatory Efficiency and performance diagnoses named Masticatory Efficiently and Performance Assessment Technique (MEPAT). With this, the proposed solution explores the possibility to standardize the information resulting from a calibration execution following these objectives:

1. To be written in a comprehensive markup language.
2. To contain relevant information about the calibration experiment.
3. To contain all the information needed to execute a diagnosis over a new specimen.
4. To be portable between different users and devices.
5. To provide scalability for new feature extraction procedures.
6. To be open-source.

**Fig 4:**
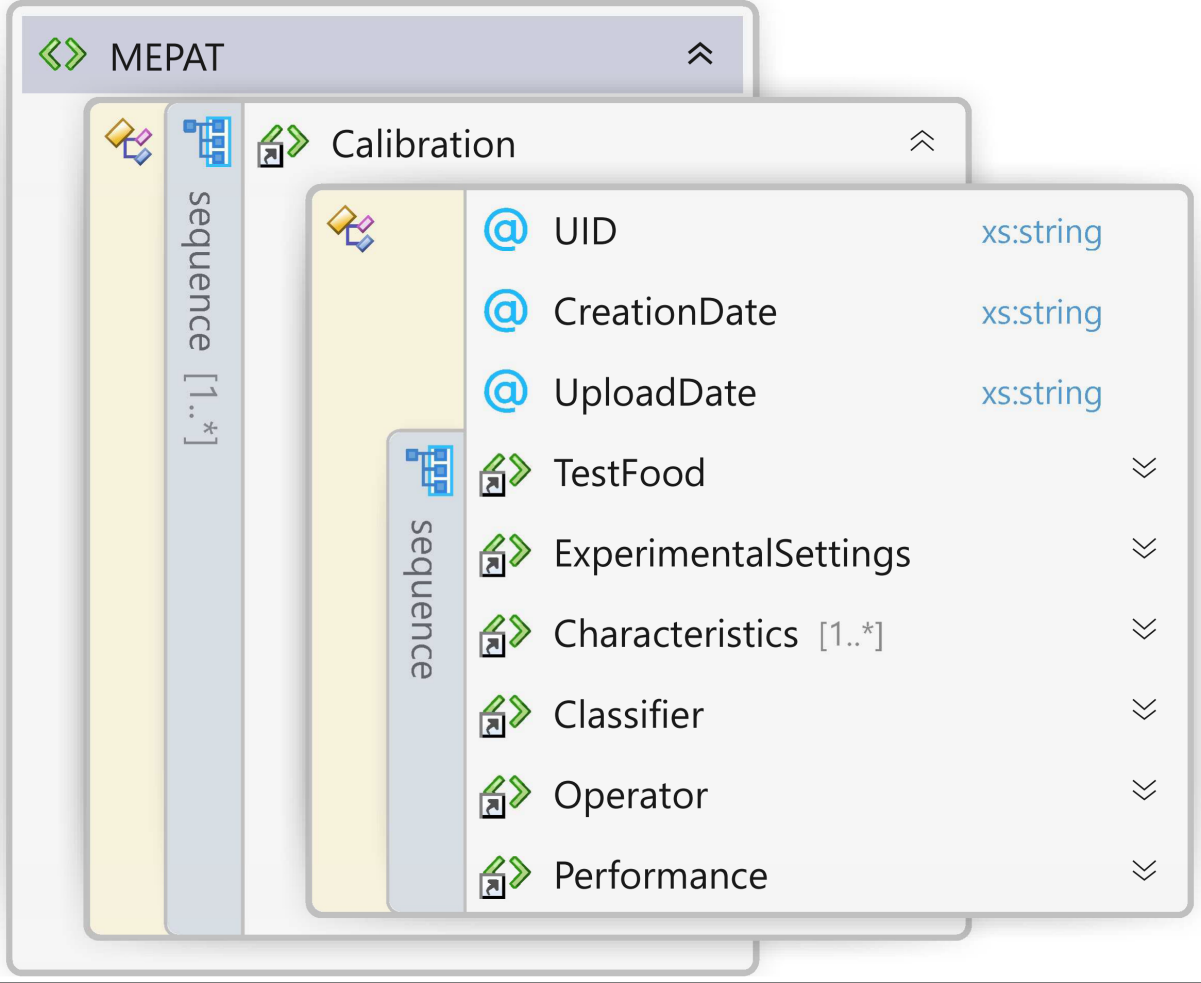
Graphical representation of the MEPAT XML schema.

The MEPAT follows a custom-made XML structure (see S1 File), which is software-independent, and can be easily stored and transferred. The MEPAT can be graphically represented as shown in Fig 4; and mathematically consists of a tuple:

MEPAT=〈TF,ES,CH,CLS,OP,PER〉 (8)

where TF represents the information about the test-food, ES represents the Experimental Settings, CH represents the set of selected features that characterize the test-food, CLS represents the trained classifier, OP represents the information about the Operator that orchestrated the calibration stage, and PER represents the performance of the overall MEPAT. On the other hand, additional information such as a Unique Identifier (UID), creation date, and upload date, may be used for synchronization purposes. Additional information about the MEPAT components can be found in S3 Appendix. The resultant information obtained from the calibration process was included in a MEPAT structure and used in further stages of this work.

### Diagnosis

The ultimate purpose of the calibration stage was to provide the necessary information to perform an adequate diagnosis of the masticatory function of an individual. To do so, the system employed a MEPAT to execute a *single diagnosis mixing-test*, which is defined as a calibrated methodology for assessing the masticatory function of an individual using a single specimen of a two-coloured chewing gum.

#### MEPAT selection

This experiment considered the MEPAT that was created during the previous calibration execution because it contained all the information necessary to perform single diagnosis mixing-tests with the available resources at the University of Guayaquil in Ecuador.

#### Clinical procedure and sample retrieval

A total of 40 volunteers (N_2_ = 40) were recruited for the testing group (G_2_): 21 females aged 73 ± 8.7 years; and 19 males aged 71 ± 9.1 years. Subjects were patients from the dental prosthetics clinic of the Faculty of Dentistry of the University of Guayaquil, Ecuador. The inclusion criteria were being older than 60 years old and complete edentulism. Exclusion criteria were TMJ dysfunction symptoms, orofacial pain, and severe cognitive impairment. Written informed consent was obtained from each subject after a full explanation of the research project. The Ethical Committee for Human and Animal Experimentation of the University of Guayaquil provided a formal approval for this experiment.

Four samples were obtained from each patient: first, patients received two consecutive tests without wearing dental prosthesis; then, patients received the next two tests 30 days after complete removable dentures were fitted during a follow-up appointment. The overall procedure for sample retrieval for was conducted as follows:

1. An operator provided the patient with a specimen of the selected test-food.
2. The operator instructed the patient to masticate the test-food specimen by 20 chewing strokes on the preferred chewing side (notice that the largest number of chewing cycles during the calibration stage was 20).
3. The operator monitored the patient while silently counting the number of chewing strokes.
4. The masticated specimen was retrieved when 20 chewing cycles were achieved.
5. The operator put the specimen between two sheets of transparent film intended for document lamination.
6. The operator flattened the specimen to a 1mm thick wafer using a calibrated press.
7. The pressed wafer was scanned for both sides.

#### Digital image analysis

The system segmented all the digital images obtained from the samples using the MS + DM + KM procedure (see S1 Appendix); then, the system extracted a set of features following the instructions stored in the CH component of the MEPAT.

#### Classification

The proposed system extracted a set of features from each sample following the instructions in the CLS component of the MEPAT. Then, the classifier categorised each sample in one of the classes related to a number of chewing strokes. This classification provided the number of chewing strokes that a healthy reference individual would need to achieve a similar degree of mixture, i.e. the *P* score of the sample.

#### Masticatory Efficiency quantification

The system computed the ME correspondent to each sample using Eq 1, considering *T* = 20 and the *P* score calculated in the Classification step. For instance, if a sample scored a *P* of 15 then the corresponding ME would be: ME = (15/20) × 100% = 75%.

### Statistical analysis

Statistical analyses were performed on MATLAB 2015a (The MathWorks Inc., MA, USA) using the Statistic Toolbox. First, the Mathews Correlation Coefficient [38] was used for validating the pattern identification performance for G_1_. Secondly, the inter-rater agreement (consensus) of consecutive ME measurements of the G_2_ was verified using the Cohen’s Kappa statistic, considering the initial and follow-up appointments separately [39]. Thirdly, the differences between the ME measured prior and after treatment with complete removable prostheses (first appointment and follow-up appointment respectively) were evaluated using the Wilcoxon signed-rank test, considering the highest ME value from the two measurements.

The performance of the proposed ANN classification approach was compared against a single-feature classification methodology. The goal was to verify the practicality of implementing a complex classification procedure instead of computing the ME directly from single MP measurements obtained from traditional methods. To do so we implemented a binary cascade to determine the closeness of each sample to the appropriate number of chewing strokes by computing its standard score (z-score) as the number of standard deviations by which the MP measurement is above the mean. The z-score has been used previously by Halazonetis et al. [13] to diagnose the state of the masticatory function by comparing the MP (*CVOH*, see S2 Appendix) measurements of a given sample against a known MP distribution. Mathematically, the z-score was computed as:

$$z_{i,T}=\frac{{mp}_{i}-{\bar{mp}}_{T}}{\sigma_{T}}$$ (9)

Where *z*_*i*,*T*_ is the z-score of the *i-*th sample computed for *T* chewing strokes, *mp*_*i*_ is the MP measured from a single feature, ${\bar{mp}}_{T}$ is the mean MP value computed from the Training Group, and *σ*_*T*_ is the standard deviation. Then, samples from the Testing Group were pre-classified as part to the *T* group if |*z*_*i*,*T*_| ≤ 0.25; hence, the core binary classification performance per *T* group was computed using the MCC score. Finally, the samples were classified in the *T* group that provided the lowest absolute z-score; i.e., where the MP value was closest to the mean.

## Results

### Calibration stage

This study retrieved a total of 400 specimens during the calibration stage (800 digital images). The complete calibration process required a combined total of 156.05 hours, which corresponded to 9 sessions of sample retrieval (~4 hours per session with 4 operators), and one session of image processing and classifier training (3.86 hours) which was performed on an Intel Core i7-5930K PC with 32GB of RAM. The Table 2 provides detailed information about the time required for the calibration phase; in this regard, the mastication, resting and digitization, and image processing steps accounted for 64.5%, 33%, and 2.5% of the calibration execution time respectively.

**Table 2:** Distribution of time shares of the calibration stage for 400 samples.

	Execution time per sample in seconds: average (std. dev.)	Total execution time in hours	Time share
**Mastication**	90.6 (19.1)	100.7	64.5%
**Digitization** ^**a**^	52.9 (4.2)	51.5	33.0%
**Image processing**	3.5 (1.1)	3.9	2.5%
**Total**	178.6 (5.8)	156.1	100.0%

^a^ Also includes the resting time

A total of 35 features with a *q* score above 0.5 were selected as good mixture characterizers (calculated with Eq 3). On every case, the Kolmogorov-Smirnov test confirmed the normality of the distribution per chewing stroke (p < 0.05); significant differences among mixing states were confirmed with ANOVA corrected with post hoc Bonferroni (p < 0.05); and showed moderate-to-high correlation with the number of chewing strokes (| ρ | > 0.5; p < 0.05). The PCA executed over the set of selected features indicated that 3 principal components (PC1 to PC3) explained 91.301%, 4.488%, and 4.211% of the variance respectively. The Table 3 lists the selected features along their corresponding *q* scores and PCA factors rotated with *Promax* rotation method.

**Table 3:** List of features selected as mixture state characterizers, showing the model, the colour space channel, the relevancy *q* score, and the PCA factors for the first 3 principal components.

Feature extraction model	Channel	*q*	PC1	PC2	PC3
A. Variance of the histogram	H	0.841	0.99	-0.02	-0.04
Entropy of the histogram	H	0.827	0.01	0.00	0.00
Value of the 1st peak of the histogram	u	0.825	0.00	0.35	0.00
Value of the 1st peak of the histogram	B	0.820	0.00	0.00	0.08
A. Variance of the pixels	G	0.805	0.00	0.00	0.00
Value of the 1st peak of the histogram	S	0.802	0.00	0.00	0.00
Value of the 1st peak of the histogram	Rn	0.800	0.00	0.00	0.00
A. Variance of the pixels	u	0.799	0.00	0.00	0.00
A. Variance of the pixels	S	0.795	0.11	0.01	0.19
A. Variance of the pixels	Rn	0.786	0.00	0.00	0.00
A. Variance of the pixels	Gn	0.783	0.70	0.26	0.10
A. Variance of the pixels	L	0.776	0.00	0.00	0.00
Value of the 1st peak of the histogram	G	0.774	0.00	0.00	0.00
Value of the 1st peak of the histogram	Gn	0.772	0.01	-0.05	0.25
A. Variance of the pixels	B	0.772	0.01	-0.06	0.27
Skewness of the histogram	H	0.769	0.00	-0.01	0.28
Mean of the pixels	H	0.706	0.00	0.00	0.00
Value of the 1st peak of the histogram	L	0.705	0.00	0.00	0.00
Value of the 1st peak of the histogram	Bn	0.692	0.00	0.00	0.00
A. Variance of the pixels	Bn	0.671	-0.01	-0.05	0.61
Entropy of the histogram	R	0.657	0.00	0.00	0.00
Entropy of the histogram	I	0.634	0.51	0.00	0.00
A. Variance of the histogram	R	0.625	-0.02	-0.03	0.41
Entropy of the histogram	u	0.625	-0.02	-0.08	0.40
Entropy of the histogram	G	0.624	-0.03	-0.05	0.46
A. Variance of the histogram	B	0.623	0.00	0.00	0.00
A. Variance of the histogram	G	0.604	0.00	0.00	0.00
Entropy of the histogram	S	0.600	0.00	0.00	0.00
A. Variance of the histogram	I	0.599	-0.03	0.23	0.32
Entropy of the histogram	L	0.598	0.00	0.00	0.00
Entropy of the histogram	B	0.595	0.00	0.00	0.00
A. Variance of the histogram	S	0.590	-0.02	0.93	-0.10
A. Variance of the histogram	L	0.576	0.00	0.00	0.00
Entropy of the histogram	Gn	0.571	0.00	0.01	0.00
Entropy of the histogram	Rn	0.558	0.00	0.00	0.74

Classifiers trained with all the 35 selected features performed slightly better than those trained solely with the first 3 principal components after 200 iterations, although no significant differences were found between training groups (*p* = 0.412). The classifier that showed the best overall performance was trained using all the features listed in Table 3, with 16 neurons in the hidden layer (*h* = 16), and was obtained after 110 iterations. Further performance details of the resultant classifier are listed in Table 4.

**Table 4:** Performance details derived from the confusion matrix of the trained best trained classifier obtained from the calibration stage.

Confusion matrix component	Score
Sensitivity	0.98
Specificity	0.99
Accuracy	0.99
MCC	0.97

On the other hand, the core performances of single-feature classifiers are detailed in S4 Appendix. The name of each features is expressed using the corresponding Mixture Feature Code (MFC, see S3 Appendix). The MCC score was computed per group to better visualize the ability of the classifier to differentiate between numbers of chewing strokes. Finally, the global classification score (including T = 0, … 20) is presented.

### Diagnosis stage

The complete diagnosis stage required a combined total of 6.23 hours, which corresponded to two sessions of sample retrieval (~2.6 hours per session with 4 operators), and two sessions of image processing and automatic classification (~ 12 minutes); with an average execution time per patient of 4.5 minutes. Some operators reported difficulties while counting the chewing cycles; in those cases, the operator asked an assistant for help and the final number of chewing cycles was determined by agreement.

The Cohen’s Kappa statistic showed that repeated measurements of ME for the G_2_ group showed almost perfect agreement, considering pre- and post-treatment appointments separately (κ ≥ 0.95). Furthermore, a Wilcoxon signed-rank test showed that a complete denture treatment for edentulous patients elicited a statistically significant increase in the ME measurements of the individuals (*Z* = -2.31, p < 0.01). Additionally, the Absolute Variance of the Histogram of the Hue channel (VhH) was evaluated separately for comparison reasons [11]; in this regard, complete denture treatment for edentulous patients elicited a statistically significant increase in VhH measurements (*p* < 0.001). The mean, median, and standard deviations of ME and VhH measurements are listed in Table 5 along with the corresponding linguistic tag associated to the ME level (see Table 1).

**Table 5:** Masticatory Efficiency (ME) and absolute variance of the histogram of the Hue (VhH) of edentulous individuals measured prior and after treatment with complete dentures.

	Statistic	ME	ME level tag	VhH
**Prior treatment**	Mean	0.26	Impeded	10.26×10^6^
	Median	0.25	Impeded	9.56×10^6^
	Standard deviation	0.22	-	1897.01
	Mode	0.50	Limited	10.11×10^6^
**After treatment**	Mean	0.71	Adequate	24.05×10^6^
	Median	0.75	Adequate	23.49×10^6^
	Standard deviation	0.23	-	3314.78
	Mode	0.75	Adequate	23.99×10^6^

## Discussion

This paper introduced new definitions for ME and MP. The differences between ME and MP were clearly distinguished, and the MP was used as a component for the calculation of the ME. Additionally, a reference scale for the ME is presented for the first time.

The calibration stage required most of the experimental time and resources, as it involved a complex (yet easier than traditional) clinical execution. On the other hand, the average execution time needed to obtain a full ME diagnosis from a patient was 5 minutes, which is considerably fast for a clinical setting. The disparity between calibration and diagnosis stages was predictable, as the calibration stage required more samples and more complex computational processing steps.

The visual features selected in this experiment as characterizers of the mixture were computed using various deterministic algorithms applied over a broad range of colour channels. Two feature reduction approaches were followed, first, a relevance-based method selected a total of 35 bare features, implying that the mixture may be assessed by more than a single feature, and that some features are better than others at characterizing the mixture. The variance of the histogram of the Hue channel of the HSI colour space provided the best overall relevancy, while the entropy of the histogram of the normalized-red channel of the normalized RGB colour space provided the lowest acceptable relevancy. On the other hand, a PCA approach selected 3 PCs that accounted for 99.9% of the variance, thus suggesting that most of the features provided little-to-none new variance information to the model.

For both feature selection approaches pattern identification and classifier training showed very high performance scores, with an MCC = 0.97 for the best case using the 35 bare features as inputs. This suggest that the proposed expert system was reliable at identifying mixture patterns for the selected test-food. On the other hand, results at S4 Appendix show that single-feature classifiers performed poorly in comparison to ANN-based classifiers for this purpose. The best single-feature classifier employed the EhH feature as the MP indicator with global MCC = 0.321. However, it is interesting to notice that many single-feature classifiers provided good core classification performance when tasked to identify samples masticated for 20 chewing strokes (T = 20); in this case Nhu, NhB, NhGn, Ehu, EhGn, VhGn, P2H, NhRn, NhL, NhG, EhH, V1H, NhS, NhI, Nhv, VhB scored an MCC ≥ 0.5, where the Nhu obtained the highest core classification performance score with MCC = 0.660.

Diagnosis stage results showed that ME of edentulous individuals were significantly higher after receiving treatment with complete dentures (p < 0.01), implying an increase in the mastication process outcome from “impeded” to “adequate” (see Table 1).

The proposed methodology can be improved in future works by strengthening the feature extraction and selection processes; as one of the key factors when computing the ME via pattern recognition is the quality of the MP indicators and the amount of relevant information that these provide to the model. Therefore, more sophisticated mixture quantification approaches such as texture analysis and wavelet transformations may significantly improve the performance of the proposed system. Also, better feature selection approaches are may help to reduce the necessity of large datasets of samples during the training stage.

It is possible to sustain that efforts to standardize MP evaluation in a worldwide scale may not be viable, as there are significant differences in digitization equipment and test-food availability among countries. Differences in the digitization equipment may produce undesirable effects on the classifier outcome, although this phenomenon has not been profoundly studied, and the proposed calibration model can be easily adapted to handle different kinds of digitization devices. Additionally, choosing the right test-food was a crucial task, as it greatly influences the outcome of the masticatory assessment [14]. A very useful list of specifications for specimens aimed to use for a two-colour-mixing ability test was presented by Schimmel, et al (2015) [9]. Regarding this, we recommend that specimens must have two different colours that would mix when chewed, be sugar-free, be easy to chew, must not have a hard coating, and should not stick to artificial dentures. Specially made test-foods for masticatory performance assessment made by Lotte® (Lotte Co., Ltd., Tokyo, Japan) may be acquired in Japan and Korea, but they are not available worldwide. Nonetheless, we consider plausible to find suitable specimens for mixing-tests most of the time.

Further applications of the proposed expert system may be executed in two different scenarios: the calibration stage should be performed within a research context with more resources, and the diagnosis stage may be performed in a clinical practice context, linked by the sharing of experimental information in the form of a MEPAT.

### Shortcomings of the proposed method

This study considered only one colour combination and brand of chewing gums, hence further studies may include other colour combinations and brands available in other regions and countries. In addition, the study sample for the diagnosis stage should be extended to include more masticatory-compromising pathologies and treatments. In our case, treatment with complete oral dentures was chosen because it represents a large portion of the daily routine of dental practice in Ecuador, and Masticatory Efficiency evaluation provides useful diagnostic information and relevant data for treatment enhancing.

It is important to notice that the proposed system involved algorithms designed to reduce the influx of the operator during the image processing steps; therefore, subsequent analysis of the same set of images will always provide the same feature measurements. Nevertheless, pattern identification involved an aleatory component, which was required to ensure the robustness of the classifier. This means that subsequent executions of the machine learning step would provide different results each time. In this experiment, the classifier validation step diminished the randomness of the calibration execution by requiring a high MCC score and the selection of the classifier with the best performance.

The proposed methodology comprises only simple pixel and histogram feature extraction models, so certain chewing gum colour combinations can affect the quality of the features. This phenomenon has been registered by Halazonetis et al. where the Circular Variance of the Hue channel performed poorly with colour combinations that included similar HUE values although easily differentiable by direct observation [13]. In this regard, the present study employed red and white chewing gum samples; so, the mixture of these two colours produced pink shades that may have affected the performance of HUE-based features.

## Conclusions

Within the limitations of this study we conclude that the proposed expert system proved able and reliable to accurately identify patterns in mixture and subsequently classify masticated two-coloured chewing gum specimens into the corresponding group represented by the number of chewing cycles applied considering a healthy reference population. Furthermore, the expert system proved able to identify differences in the ME of edentulous individuals with and without total prosthesis. Finally, we propose the inclusion of the newly presented ME definition and reference scale in further research studies in this field.

## Supporting information

S1 Appendix Border-preserving region segmentation and classification. (DOCX)

S2 Appendix Feature extraction models. (DOCX)

S3 Appendix Detailed information about the MEPAT construction. (DOCX)

S4 Appendix Core classification performance of single-feature classifiers. (DOCX)

S1 File XML schema for the MEPAT. (XML)

## References

[1] Ohira A, Ono Y, Yano N, Takagi Y. The effect of chewing exercise in preschool children on maximum bite force and masticatory performance. Int J Paediatr Dent. 2012;22: 146–53. doi: 10.1111/j.1365-263X.2011.01162.x 21781200 10.1111/j.1365-263X.2011.01162.x

[2] Bates J, Stafford G, Harrison A. Masticatory function—a review of the literature. III. Masticatory performance and efficiency. J Oral Rehabil. 1976;3: 57–67. Recuperado: http://onlinelibrary.wiley.com/doi/10.1111/j.1365-2842.1976.tb00929.x/full 772184 10.1111/j.1365-2842.1976.tb00929.x

[3] The Glossary of Prosthodontic Terms. J Prosthet Dent. Elsevier; 2005;94: 10–92. doi: 10.1016/j.prosdent.2005.03.013 16080238 10.1016/j.prosdent.2005.03.013

[4] Dai R, Lam OLT, Lo ECM, Li LSW, Wen Y, McGrath C. Orofacial functional impairments among patients following stroke: a systematic review. Oral Dis. 2014; doi: 10.1111/odi.12274 25041135 10.1111/odi.12274

[5] Yamashita S, Hatch JP, Rugh JD. Does chewing performance depend upon a specific masticatory pattern? J Oral Rehabil. 1999;26: 547–53. Recuperado: http://www.ncbi.nlm.nih.gov/pubmed/10445472 10445472 10.1046/j.1365-2842.1999.00446.x

[6] Gil-Montoya JA, Subirá C, Ramón JM, González-Moles MA. Oral health-related quality of life and nutritional status. J Public Health Dent. 2008;68: 88–93. doi: 10.1111/j.1752-7325.2007.00082.x 18248335 10.1111/j.1752-7325.2007.00082.x

[7] Manly RS, Braley LC. Masticatory Performance and Efficiency. J Dent Res. 1950;29: 448–462. doi: 10.1177/00220345500290040701 15436916 10.1177/00220345500290040701

[8] Schimmel M, Christou P, Herrmann F, Müller F. A two-colour chewing gum test for masticatory efficiency: development of different assessment methods. J Oral Rehabil. 2007;34: 671–8. doi: 10.1111/j.1365-2842.2007.01773.x 17716266 10.1111/j.1365-2842.2007.01773.x

[9] Schimmel M, Christou P, Miyazaki H, Halazonetis D, Herrmann FR, Müller F. A novel colourimetric technique to assess chewing function using two-coloured specimens: validation and application. J Dent. 2015;43: 955–964. doi: 10.1016/j.jdent.2015.06.003 26111925 10.1016/j.jdent.2015.06.003

[10] Weijenberg RAF, Scherder EJA, Visscher CM, Gorissen T, Yoshida E, Lobbezoo F. Two-colour chewing gum mixing ability: digitalisation and spatial heterogeneity analysis. J Oral Rehabil. 2013;40: 737–43. doi: 10.1111/joor.12090 23927753 10.1111/joor.12090

[11] Vaccaro G, Pelaez JI, Gil JA. Choosing the best image processing method for masticatory performance assessment when using two-coloured specimens. J Oral Rehabil. 2016;43: 496–504. doi: 10.1111/joor.12392 26968333 10.1111/joor.12392

[12] Prinz JF. Quantitative evaluation of the effect of bolus size and number of chewing strokes on the intra-oral mixing of a two-colour chewing gum. J Oral Rehabil. 1999;26: 243–7. 10194734 10.1046/j.1365-2842.1999.00362.x

[13] Halazonetis DJ, Schimmel M, Antonarakis GS, Christou P. Novel software for quantitative evaluation and graphical representation of masticatory efficiency. J Oral Rehabil. 2013;40: 329–35. doi: 10.1111/joor.12043 23452188 10.1111/joor.12043

[14] van der Bilt A, Mojet J, Tekamp FA, Abbink JH. Comparing masticatory performance and mixing ability. J Oral Rehabil. 2010;37: 79–84. doi: 10.1111/j.1365-2842.2009.02040.x 19968766 10.1111/j.1365-2842.2009.02040.x

[15] van der Bilt A. Assessment of mastication with implications for oral rehabilitation: a review. J Oral Rehabil. 2011;38: 754–80. doi: 10.1111/j.1365-2842.2010.02197.x 21241351 10.1111/j.1365-2842.2010.02197.x

[16] Beucher S, Lantuéjoul C. Use of Watersheds in Contour Detection. International workshop on image processing, real-time edge and motion detection. 1979.

[17] Lu S, Wang S, Zhang Y. A note on the marker-based watershed method for X-ray image segmentation. Comput Methods Programs Biomed. 2017;141: 1–2. doi: 10.1016/j.cmpb.2017.01.014 28241959 10.1016/j.cmpb.2017.01.014

[18] Zhang Y, Dong Z, Phillips P, Wang S, Ji G, Yang J. Exponential Wavelet Iterative Shrinkage Thresholding Algorithm for compressed sensing magnetic resonance imaging. Inf Sci (Ny). 2015;322: 115–132. doi: 10.1016/j.ins.2015.06.017

[19] Comaniciu D, Meer P. Mean shift: a robust approach toward feature space analysis. IEEE Trans Pattern Anal Mach Intell. IEEE; 2002;24: 603–619. doi: 10.1109/34.1000236

[20] Christoudias CM, Georgescu B, Meer P. Synergism in low level vision. Object recognition supported by user interaction for service robots. IEEE Comput. Soc; 2002 pp. 150–155. doi: 10.1109/ICPR.2002.1047421

[21] MacQueen J. Some methods for classification and analysis of multivariate observations Proceedings of the Fifth Berkeley Symposium on Mathematical Statistics and Probability, Volume 1: Statistics. The Regents of the University of California; 1967.

[22] Commission Internationale de L’Eclairage. Publication No. CIE 15.2. Colorimetry. 2nd ed. Vienna, Austria: Central Bureau of the CIE.; 1986;

[23] Zhang Y, Wang S, Phillips P, Ji G. Binary PSO with mutation operator for feature selection using decision tree applied to spam detection. Knowledge-Based Syst. 2014;64: 22–31. doi: 10.1016/j.knosys.2014.03.015

[24] Veredas F, Mesa H, Morente L. Binary tissue classification on wound images with neural networks and bayesian classifiers. IEEE Trans Med Imaging. IEEE; 2010;29: 410–27. doi: 10.1109/TMI.2009.2033595 19825516 10.1109/TMI.2009.2033595

[25] Judd DB. Hue Saturation and Lightness of Surface Colors with Chromatic Illumination. J Opt Soc Am. Optical Society of America; 1940;30: 2 doi: 10.1364/JOSA.30.000002

[26] Vezhnevets V, Sazonov V, Andreeva A. A Survey on Pixel-Based Skin Color Detection Techniques. Graphicon-2003. Moscow, Russia; 2003. pp. 85–92.

[27] Jolliffe I. Principal Component Analysis. New York: Springer-Verlag; 2002 doi: 10.1007/b98835

[28] Haykin S. Neural Networks: A Comprehensive Foundation. Prentice Hall PTR; 1998;

[29] Ripley B.D. Pattern Recognition and Neural Networks. Oxford, UK.: Cambridge University Press; 1996.

[30] Zhang Y, Sun Y, Phillips P, Liu G, Zhou X, Wang S. A Multilayer Perceptron Based Smart Pathological Brain Detection System by Fractional Fourier Entropy. J Med Syst. Springer US; 2016;40: 173 doi: 10.1007/s10916-016-0525-2 27250502 10.1007/s10916-016-0525-2

[31] Wang S-H, Zhang Y, Li Y-J, Jia W-J, Liu F-Y, Yang M-M, et al Single slice based detection for Alzheimer’s disease via wavelet entropy and multilayer perceptron trained by biogeography-based optimization. Multimed Tools Appl. Springer US; 2016; 1–25. doi: 10.1007/s11042-016-4222-4

[32] Suzuki K, editor. Artificial Neural Networks—Architectures and Applications. InTech; 2013 doi: 10.5772/3409

[33] Rumelhart DE, Hinton GE, Williams RJ. Learning representations by back-propagating errors. Nature. 1986;323: 533–536. doi: 10.1038/323533a0

[34] Čepek M, Šnorek M, Chudáček V. Ecg signal classification using game neural network and its comparison to other classifiers. Artificial Neural Networks-ICANN 2008. Springer; 2008 pp. 768–777.

[35] Callejón AM, Casado AM, Fernández MA, Peláez JI. A System of Insolvency Prediction for industrial companies using a financial alternative model with neural networks. Int J Comput Intell Syst. Taylor & Francis Group; 2013;6: 29–37. doi: 10.1080/18756891.2013.754167

[36] Vaccaro G, Pelaez JI. Dental tissue classification using computational intelligence and digital image analysis Biodental Engineering III—Proceedings of the 3rd International Conference on Biodental Engineering, BIODENTAL 2014. Taylor and Francis—Balkema; 2014 pp. 221–226. doi: 10.1201/b17071

[37] Lawrence S, Giles CL, Tsoi AC. Lessons in neural network training: Overfitting may be harder than expected Proceedings of the Fourteenth National Conference on Artificial Intelligence, AAAI-97. Menlo Park, California: AAAI Press; 1997 pp. 540–545.

[38] Matthews BW. Comparison of the predicted and observed secondary structure of T4 phage lysozyme Biochim Biophys Acta—Protein Struct. 1975;405: 442–451. doi: 10.1016/0005-2795(75)90109-9 10.1016/0005-2795(75)90109-9 1180967

[39] Cohen J. A Coefficient of Agreement for Nominal Scales. Educ Psychol Meas. 1960;20: 37–46. doi: 10.1177/001316446002000104

