# Supplementary material for: A novel expert system for objective masticatory efficiency assessment
Source: PLoS One. 2018 Jan 31;13(1):e0190386. doi: 10.1371/journal.pone.0190386 (PMC5791957; doi:10.1371/journal.pone.0190386)
Supplement: S1 Appendix — (DOCX) [file pone.0190386.s001.docx]

**S1 Appendix. Border-preserving region segmentation and classification**

The MS algorithm is based on kernel density estimation, doesn’t assume a predefined morphology in the image, and has been proved efficient for automatic, edge-preserving segmentation of colour images. The procedure has been thoughtfully described in previous studies. Briefly, a set of input points (in this case, pixels of an image) are regarded as random samples drawn from a probability density function (PDF). Then, the peaks of the PDF are found, which in turn correspond to the modes of the data. The data is then divided into clusters by associating each point to its mode. Mathematically, let *g*(*x*) be a radially symmetric kernel function, *h* be a user controlled parameter defining a window radius, and *x_i_*, *i* = 1,…,*n* a set of points in the *d*-dimensional space *R^d^*. For each point *x_k_* (1 ≤ *k* ≤ *n*), the sequence of successive locations of the kernel *y_j_*, *j*=0,1,2,… is defined as follows

| $y_{j+1}=\frac{\sum_{i=1}^{n} x_{i}g\left( \left\Vert\frac{y_{j}-x_{i}}{h} \right\Vert^{2} \right)}{\sum_{i=1}^{n} g\left( \left\Vert\frac{y_{j}-x_{i}}{h} \right\Vert^{2} \right)}$ | (1) |
| --- | --- |

where, *y*_0_ = *x_k_*. The Mean Shift procedure performs gradient ascent on the underlying PDF; therefore, the sequence *y_j_* converges to a mode of this function. In this paper we considered the following kernel function:

| $g\left( x \right)=\frac{1}{2}e^{-\frac{x}{2}}$ | (2) |
| --- | --- |

The task of classifying the segmented clusters between those corresponding to the bolus versus the background of the image was fulfilled by a parametric K-Means procedure. It aimed to classify the regions of the image in two groups: the bolus located in the centre of the image, which is considered as the Region of Interest (ROI); and the Background, surrounding the bolus. The Background is characterized for its relative uniformity in relation to the bolus, but in some cases a handwritten label or a sticker may be present.

During empirical experimentation we noticed that in many occasions the colours of some parts of the bolus were more similar to the Background than the rest of the bolus; thus affecting the correctness of the masking and region classification using K-Means. Therefore, we introduced a custom pre-processing step where a distance map image was created from the softened/clustered image, and taking into account the corners as known Background regions. The final region classification process was constructed as follows:

1. Stablish the mean background colour (*b*) as the mean colour of the super-pixels (clusters) resultant from the MS segmentation that are located in the four corners of the image, where *b* is in the CIE Lab colour space.
2. For each pixel *x_i_* of the segmented image, compute the logarithm of the colour Euclidean distance (*d_i_*) to *b* (*d_i_*=log ||*x_i_*-*b*||) and form a new DM image. This reduces the possible relative difference between regions of the bolus and preserves the homogeneity of the Background.
3. Classify the pixels in the DM in two clusters (*k*=2) using the KM procedure.
4. Select the position of the pixels in the cluster located in the centre of the image as the ROI.
5. Mask and extract the information of the pixels corresponding to the ROI in the original image.
