# Supplementary material for: A novel expert system for objective masticatory efficiency assessment
Source: PLoS One. 2018 Jan 31;13(1):e0190386. doi: 10.1371/journal.pone.0190386 (PMC5791957; doi:10.1371/journal.pone.0190386)
Supplement: S2 Appendix — (DOCX) [file pone.0190386.s002.docx]

**S2 Appendix. Feature extraction models**

- The mean of the pixels values (*M*), representing the average colour, computed as:

| $M=\mu=\frac{\sum x_{i}}{A}$ | (1) |
| --- | --- |

where *x_i_* is the *i*^th^ pixel, and *A* is the total number of pixels in the region of interest (ROI).

- The absolute variance of the pixels values (*V*), representing the spread of colours, computed as:

| $V=\frac{\sum\left\vert x_{i}- \mu\right\vert}{A-1}$ | (2) |
| --- | --- |

- The absolute variance of the histogram (*Vh*), representing the spread of the histogram, computed as:

| $Vh=\sigma_{h}=\frac{\sum\left\vert H- \mu_{h} \right\vert}{99}$ | (3) |
| --- | --- |

where $\mu_{h}$ is the mean of the Histogram function $H$.

- The skewness of the histogram (*Sh*), measuring the asymmetry of the histogram, computed as:

| $Sh=\sum_{i=1}^{100} \frac{{(h_{i}-\mu_{h})}^{3}}{99 \times{\sigma_{h}}^{3}}$ | (4) |
| --- | --- |

where $h_{i}$is the *i*^th^ histogram bin.

- The energy of the histogram (*Eh*), that is a discriminative representation of the colour distribution, computed as:

| $Eh=\sum_{i=1}^{100} h_{i}^{2}$ | (5) |
| --- | --- |

- The entropy of the histogram (*Nh*), that is an estimator of the randomness of the colour distribution, computed as:

| $Nh=-\sum_{i=1}^{100} h_{i}\times\log_{2} (h_{i})$ | (6) |
| --- | --- |

- And the position (P1, P2) and height (T1, T2) of the two highest peaks of the histogram (local maxima), bearing in mind its application in previous works [20,35].

Additionally, we also consider the circular variance of the pixels of the Hue channel of the HSI colour space (*CVOH*), computed as:

| $CVOH=1-\frac{1}{A}\sqrt{\left( \sum_{i=1}^{A} \cos\left( \theta_{i} \right) \right)^{2}+\left( \sum_{i=1}^{A} \sin\left( \theta_{i} \right) \right)^{2}}$ | (7) |
| --- | --- |

where $\theta_{i}$ is the Hue value, and $A$ is the total number of pixels in the ROI.
