# Supplementary material for: A novel expert system for objective masticatory efficiency assessment
Source: PLoS One. 2018 Jan 31;13(1):e0190386. doi: 10.1371/journal.pone.0190386 (PMC5791957; doi:10.1371/journal.pone.0190386)
Supplement: S3 Appendix — (DOCX) [file pone.0190386.s003.docx]

**S3 Appendix. Detailed information about the MEPAT construction**

The TF component, comprises information about the Test-Food type (chewing gum or paraffin), the brand, colours, origin, and additional details; intended for accurate replication of the specimen formation and structural characteristics among different clinical settings, operators, and locations.

The ES component, comprises information about the experimental settings and chewing cycles involved during calibration: the “Name” attribute refers to a custom and comprehensive name that can be given to the calibration experiment, the “NumberOfPatients” contains the number of patients involved in the experiment, the “ChewingCycles” component refers to the set of chewing strokes considered (the ***C*** set), the “EvaluationCycles” component allows to assign a custom number of evaluation cycles although we consider 20 chewing strokes as the optimal number of evaluation cycles; finally, the “ClinicalProcedure” component contains the detailed procedure for sample retrieval, handling, and processing, intended for accurate reproduction of the mixing test.

The CH component, comprises information about the features that best characterize the sample. It is composed by a sequence of “Feature” tags, where each one includes an “Index” attribute that represents its ordering position, an “MFC” attribute standing for Mixture Feature Code, and a “Description” attribute. We have defined the MFC as a unique name that identifies features used for mixture quantification. In the context of this study, features were labelled with a unique MFC (see Table 1), which considers all of the 121 features obtained from 10 general image processing methods applied over 12 different channels (considering 4 colour spaces), and the circular variance of the hue channel (for legacy and comparison purposes). In the case that new features are added to the proposed model, these should be assigned a new and unique MFC. On the other hand, the “Optimal” attribute indicates the MFC codes of the feature that achieved the highest *q*-score, thus being considered the optimal MP indicator in this specific case.

The CLS component, comprises information about the trained classifier, which in this case is an ANN modelled as a multilayer perceptron. The ANN structure is composed by a sequence of “Layer” components, representing the Input, Hidden, and Output layers of the network; then, each “Layer” is composed by a sequence of “Neuron” components (ordered by an “Index” attribute”), which are subsequently composed by a sequence of “Weight” components (also ordered by an “Index” attribute). A fully functional classifier can be constructed using the information provided by the CLS component, provided that the inputs correspond to the same set of characteristics used in the calibration stage.

The OP component, comprises information about the head clinician Operator that orchestrated the calibration stage, intended for contact purposes.

Finally, the PER component, comprises information about the Sensitivity, Specificity, and Accuracy of the resultant classifier, thus representing the overall performance of the MEPAT.

**Table 1. Assignation of Mixture Feature Codes (MFC) to the image processing methods included in the proposed model as feature quantification indicators.**

| Visual feature extraction model | Colour space components | | | | | | | | | | | |
| --- | --- | --- | --- | --- | --- | --- | --- | --- | --- | --- | --- | --- |
|  | RGB | | | CIE L*u*v* | | | HSI | | | Normalized RGB | | |
|  | R | G | B | L | u | v | H | S | I | Rn | Gn | Bn |
| Mean of the pixels values | MR | MG | MB | ML | Mu | Mv | MH | MS | MI | MRn | MGn | MBn |
| Absolute Variance of the pixels values | VR | VG | VB | VL | Vu | Vv | VH | VS | VI | VRn | VGn | VBn |
| Position and values of the 1^st^ and 2^nd^ highest histogram peaks | P1R P2R  V1R  V2R | P1G P2G  V1G  V2G | P1B P2B  V1B  V2B | P1L P2L  V1L  V2L | P1u P2u  V1u  V2u | P1v P2v  V1v  V2v | P1H P2H  V1H  V2H | P1S P2S  V1S  V2S | P1I P2I  V1I  V2I | P1Rn P2Rn  V1Rn  V2Rn | P1Gn P2Gn  V1Gn  V2Gn | P1Bn P2Bn  V1Bn  V2Bn |
| Absolute Variance of the histogram | VhR | VhG | VhB | VhL | Vhu | Vhv | VhH | VhS | VhI | VhRn | VhGn | VhBn |
| Skewness of the histogram | ShR | ShG | ShB | ShL | Shu | Shv | ShH | ShS | ShI | ShRn | ShGn | ShBn |
| Energy of the histogram | EhR | EhG | EhB | EhL | Ehu | Ehv | EhH | EhS | EhI | EhRn | EhGn | EnBn |
| Entropy of the histogram | NhR | NhG | NhB | NhL | Nhu | Nhv | NhH | NhS | NhI | NhRn | NhGn | NhBn |
| Circular variance | - | - | - | - | - | - | CVOH | - | - | - | - | - |

The MFC has been constructed by relating feature extraction models to the corresponding colour space components.
