# Supplementary material for: A novel expert system for objective masticatory efficiency assessment
Source: PLoS One. 2018 Jan 31;13(1):e0190386. doi: 10.1371/journal.pone.0190386 (PMC5791957; doi:10.1371/journal.pone.0190386)
Supplement: S4 Appendix — (DOCX) [file pone.0190386.s004.docx]

**S4 Appendix.** **Core classification performance of single-feature classifiers.**

| MFC Code | MCC score per group of chewing strokes (T) | | | | | |
| --- | --- | --- | --- | --- | --- | --- |
|  | T = 0 | T = 5 | T = 10 | T = 15 | T = 20 | Global |
| MR | 0.036 | -0.127 | -0.193 | 0.016 | 0.012 | 0.069 |
| VR | 0.088 | 0.143 | 0.129 | 0.138 | 0.091 | 0.114 |
| P1R | -0.167 | -0.107 | -0.161 | -0.018 | 0.060 | -0.080 |
| P2R | 0.0365 | 0.082 | 0.030 | -0.119 | 0.056 | 0.057 |
| V1R | -0.002 | -0.165 | -0.075 | -0.211 | 0.452 | 0.081 |
| V2R | -0.002 | -0.101 | 0.129 | 0.129 | -0.193 | 0.062 |
| VhR | 0.170 | -0.246 | -0.154 | -0.260 | 0.454 | 0.238 |
| ShR | 0.170 | 0.091 | 0.167 | 0.084 | 0.084 | 0.113 |
| EhR | 0.060 | -0.036 | -0.020 | 0.055 | 0.256 | 0.057 |
| NhR | 0.170 | 0.212 | -0.154 | -0.276 | 0.442 | 0.232 |
| MG | -0.049 | 0.382 | 0.528* | -0.004 | 0.321 | 0.107 |
| VG | -0.070 | -0.006 | 0.133 | -0.107 | -0.030 | 0.045 |
| P1G | -0.070 | -0.147 | -0.054 | 0.091 | 0.256 | 0.105 |
| P2G | -0.070 | -0.070 | 0.016 | -0.154 | -0.139 | 0.070 |
| V1G | -0.087 | -0.127 | 0.288 | 0.133 | -0.292 | 0.166 |
| V2G | 0.088 | -0.006 | 0.062 | -0.132 | -0.324 | -0.067 |
| VhG | 0.123 | 0.196 | -0.211 | -0.020 | 0.256 | 0.121 |
| ShG | 0.123 | 0.264 | 0.016 | -0.020 | -0.020 | 0.040 |
| EhG | 0.348 | -0.008 | 0.115 | -0.004 | 0.257 | 0.053 |
| NhG | 0.248 | 0.140 | -0.193 | -0.228 | 0.528* | 0.241 |
| MB | -0.070 | 0.382 | 0.357 | -0.077 | 0.133 | 0.158 |
| VB | 0.241 | 0.142 | 0.233 | -0.077 | -0.030 | 0.113 |
| P1B | 0.060 | -0.062 | -0.051 | 0.138 | 0.149 | 0.083 |
| P2B | -0.002 | -0.125 | 0.062 | -0.020 | -0.077 | 0.032 |
| V1B | 0.123 | 0.206 | 0.188 | 0.186 | 0.357 | 0.199 |
| V2B | -0.127 | -0.107 | 0.452 | -0.154 | -0.260 | -0.190 |
| VhB | 0.123 | 0.016 | -0.004 | -0.174 | 0.518* | 0.060 |
| ShB | -0.070 | 0.311 | -0.020 | 0.321 | -0.020 | 0.078 |
| EhB | 0.292 | -0.075 | 0.167 | 0.091 | 0.375 | 0.166 |
| NhB | 0.248 | 0.115 | -0.193 | -0.228 | 0.596* | 0.237 |
| ML | 0.123 | 0.382 | 0.528* | -0.077 | 0.091 | 0.177 |
| VL | 0.241 | -0.036 | 0.016 | -0.132 | -0.004 | 0.038 |
| P1L | 0.348 | -0.062 | 0.288 | -0.174 | -0.154 | 0.175 |
| P2L | 0.015 | -0.054 | 0.138 | -0.020 | -0.119 | 0.049 |
| V1L | -0.070 | -0.036 | 0.000 | -0.075 | -0.228 | -0.001 |
| V2L | 0.348 | -0.183 | 0.133 | 0.016 | -0.211 | 0.123 |
| VhL | 0.182 | 0.016 | -0.145 | -0.051 | 0.256 | 0.089 |
| ShL | 0.123 | 0.075 | -0.051 | -0.132 | 0.055 | 0.080 |
| EhL | 0.292 | 0.032 | 0.149 | -0.054 | 0.366 | 0.123 |
| NhL | 0.248 | 0.167 | -0.193 | -0.244 | 0.528* | 0.253 |
| Mu | 0.088 | 0.206 | 0.129 | -0.004 | 0.288 | 0.079 |
| Vu | 0.123 | 0.142 | 0.321 | -0.193 | 0.055 | 0.143 |
| P1u | 0.155 | 0.029 | -0.054 | -0.119 | -0.145 | 0.084 |
| P2u | 0.060 | 0.140 | -0.211 | 0.023 | -0.004 | 0.045 |
| V1u | 0.060 | 0.140 | -0.154 | -0.211 | -0.077 | -0.116 |
| V2u | -0.070 | -0.107 | 0.023 | -0.020 | 0.062 | -0.047 |
| Vhu | -0.049 | 0.068 | 0.084 | -0.211 | 0.233 | 0.107 |
| Shu | 0.060 | 0.228 | 0.091 | 0.055 | 0.211 | 0.107 |
| Ehu | -0.115 | -0.183 | -0.028 | 0.016 | 0.596* | 0.089 |
| Nhu | 0.292 | 0.167 | -0.051 | -0.244 | 0.660* | 0.209 |
| Mv | -0.020 | -0.054 | -0.030 | -0.174 | 0.233 | 0.067 |
| Vv | 0.182 | -0.062 | 0.000 | -0.154 | 0.149 | 0.001 |
| P1v | -0.102 | -0.107 | 0.133 | -0.051 | 0.084 | 0.091 |
| P2v | 0.248 | 0.016 | 0.055 | -0.203 | -0.228 | 0.101 |
| V1v | 0.170 | 0.075 | 0.016 | 0.016 | -0.174 | 0.056 |
| V2v | 0.170 | 0.110 | -0.154 | -0.107 | -0.154 | -0.137 |
| Vhv | 0.182 | -0.199 | -0.020 | -0.124 | 0.186 | 0.111 |
| Shv | 0.248 | 0.142 | 0.084 | -0.051 | -0.102 | 0.109 |
| Ehv | 0.170 | 0.044 | -0.020 | -0.098 | 0.355 | -0.088 |
| Nhv | 0.170 | 0.212 | -0.051 | -0.244 | 0.528* | 0.188 |
| MH | 0.000 | 0.471 | 0.257 | 0.133 | 0.357 | 0.001 |
| VH | 0.241 | -0.199 | 0.257 | 0.091 | 0.375 | 0.211 |
| P1H | 0.000 | 0.153 | 0.186 | 0.000 | 0.000 | 0.000 |
| P2H | -0.070 | 0.212 | -0.102 | -0.107 | 0.587* | 0.157 |
| V1H | 0.388 | -0.125 | 0.091 | 0.133 | 0.528* | 0.199 |
| V2H | 0.170 | -0.165 | -0.020 | 0.186 | -0.124 | 0.105 |
| VhH | 0.241 | 0.174 | -0.132 | -0.193 | 0.442 | -0.216 |
| ShH | 0.348 | -0.146 | -0.124 | 0.133 | 0.016 | 0.106 |
| EhH | 0.000 | 0.277 | 0.129 | 0.257 | 0.528* | 0.321 |
| NhH | 0.241 | 0.257 | 0.000 | -0.211 | 0.442 | 0.121 |
| MS | -0.049 | 0.206 | 0.452 | -0.004 | 0.188 | 0.083 |
| VS | -0.087 | 0.153 | 0.257 | 0.023 | 0.055 | 0.085 |
| P1S | 0.088 | 0.110 | 0.062 | -0.216 | -0.257 | -0.127 |
| P2S | 0.248 | 0.056 | 0.091 | -0.203 | 0.188 | 0.137 |
| V1S | -0.087 | -0.008 | 0.215 | -0.119 | -0.174 | -0.081 |
| V2S | -0.036 | -0.215 | 0.115 | 0.321 | -0.193 | 0.141 |
| VhS | 0.088 | 0.174 | 0.115 | -0.004 | 0.366 | 0.078 |
| ShS | -0.070 | 0.350 | -0.051 | 0.321 | -0.145 | 0.142 |
| EhS | -0.127 | 0.056 | 0.005 | 0.023 | 0.452 | 0.054 |
| NhS | 0.292 | 0.264 | -0.154 | -0.228 | 0.528* | 0.270 |
| MI | 0.292 | -0.107 | 0.149 | 0.016 | 0.200 | 0.108 |
| VI | -0.002 | 0.075 | -0.102 | 0.055 | 0.062 | 0.038 |
| P1I | -0.092 | -0.008 | -0.339 | 0.320 | -0.098 | 0.097 |
| P2I | 0.155 | -0.062 | -0.102 | -0.211 | 0.016 | 0.080 |
| V1I | -0.036 | -0.125 | -0.077 | -0.132 | 0.275 | -0.105 |
| V2I | -0.070 | -0.125 | 0.257 | 0.129 | -0.107 | 0.125 |
| VhI | 0.123 | -0.036 | -0.154 | -0.244 | 0.186 | -0.125 |
| ShI | 0.574* | 0.016 | 0.321 | 0.133 | 0.062 | 0.120 |
| EhI | -0.087 | -0.036 | -0.051 | -0.004 | 0.366 | -0.048 |
| NhI | 0.241 | 0.174 | -0.174 | -0.228 | 0.528* | 0.245 |
| MRn | 0.123 | 0.382 | 0.186 | -0.102 | 0.186 | 0.175 |
| VRn | 0.088 | 0.206 | 0.275 | -0.051 | 0.149 | 0.130 |
| P1Rn | -0.020 | -0.006 | -0.030 | -0.020 | 0.084 | 0.022 |
| P2Rn | -0.105 | 0.115 | 0.245 | 0.051 | -0.197 | 0.124 |
| V1Rn | -0.087 | 0.350 | -0.075 | 0.257 | -0.054 | 0.126 |
| V2Rn | 0.170 | -0.101 | 0.200 | -0.124 | 0.023 | 0.100 |
| VhRn | 0.170 | 0.140 | -0.184 | -0.030 | 0.357 | 0.136 |
| ShRn | -0.049 | 0.029 | 0.133 | -0.102 | -0.107 | 0.073 |
| EhRn | -0.049 | -0.165 | -0.077 | -0.020 | 0.235 | 0.078 |
| NhRn | 0.348 | 0.228 | -0.174 | -0.228 | 0.528* | 0.278 |
| MGn | 0.241 | 0.206 | 0.366 | -0.102 | 0.357 | 0.231 |
| VGn | 0.088 | 0.075 | 0.129 | 0.257 | 0.055 | 0.104 |
| P1Gn | 0.036 | -0.107 | -0.004 | 0.188 | -0.030 | 0.039 |
| P2Gn | -0.020 | 0.142 | -0.102 | 0.288 | 0.375 | 0.126 |
| V1Gn | -0.002 | -0.085 | 0.062 | 0.235 | -0.154 | 0.056 |
| V2Gn | 0.000 | 0.304 | 0.129 | -0.193 | -0.051 | 0.07 |
| VhGn | 0.123 | 0.194 | -0.174 | -0.102 | 0.587* | -0.190 |
| ShGn | -0.049 | 0.140 | 0.257 | -0.077 | -0.051 | 0.093 |
| EhGn | 0.123 | -0.165 | 0.215 | 0.257 | 0.587* | 0.231 |
| NhGn | 0.123 | 0.115 | -0.174 | -0.228 | 0.596* | 0.202 |
| MBn | 0.241 | 0.312 | 0.133 | -0.124 | 0.133 | 0.175 |
| VBn | 0.182 | 0.434 | 0.233 | 0.091 | 0.167 | 0.195 |
| P1Bn | 0.060 | 0.142 | 0.288 | 0.016 | -0.228 | 0.098 |
| P2Bn | -0.102 | 0.270 | 0.133 | -0.020 | -0.054 | 0.083 |
| V1Bn | -0.127 | -0.006 | 0.366 | 0.133 | 0.091 | 0.080 |
| V2Bn | -0.127 | -0.125 | 0.055 | -0.193 | -0.211 | 0.129 |
| VhBn | 0.088 | 0.196 | -0.211 | -0.077 | 0.257 | 0.149 |
| ShBn | 0.348 | 0.142 | 0.235 | -0.165 | -0.107 | 0.183 |
| EnBn | -0.087 | -0.127 | 0.091 | -0.054 | 0.062 | 0.080 |
| NhBn | 0.123 | 0.082 | -0.193 | -0.211 | 0.452 | 0.179 |
| CVOH | 0.241 | 0.257 | 0.000 | -0.211 | 0.442 | 0.188 |

(*) Good classification performance (|MCC| ≥ 0.5).
